# The PBL teaching method in neurology education in the traditional Chinese medicine undergraduate students: An observational study

**DOI:** 10.1097/MD.0000000000035143

**Published:** 2023-09-29

**Authors:** Yun Jin Kim

**Affiliations:** a School of Traditional Chinese Medicine, Xiamen University Malaysia, Sepang, Selangor, Malaysia.

**Keywords:** neurology education, observational study, PBL, students perspective, traditional Chinese medicine

## Abstract

The objective of this study was to investigate the impact of the problem-based learning (PBL) method on Neurology education for Traditional Chinese Medicine (TCM) undergraduate students. This observational study was conducted during the 2020/02 and 2020/04 intakes of the third year TCM undergraduate students at School of Traditional Chinese Medicine, Xiamen University Malaysia. A total of 86 students were enrolled in the study and randomly assigned to either conventional learning groups or PBL groups. Students who missed more than 1 session of the course or did not complete the questionnaires during the evaluation periods were excluded from the study (n = 0). An independent sample *t* test was used to compare the results between the 2 groups, with a significance level set as *P* < .05. The PBL group demonstrated significantly higher scores in theoretical and clinical practical examinations, satisfaction with the teaching level, students perspectives, and self-learning skills. Additionally, the PBL group had significantly higher scores on the dundee ready educational environment measure compared to students in the conventional group (*P* < .05). The implementation of the PBL teaching method in Neurology education for TCM undergraduate students proved to be an engaging and effective learning approach. It significantly improved students learning performance and their ability to analyze and solve problems related to neurology diseases and their management knowledge.

## 1. Introduction

Traditional Chinese medicine (TCM) education is an important educational component of medical educational system in Malaysia. The traditional and complementary medicine Council, Ministry of Health, and Ministry of Higher Education stipulates undergraduate TCM higher education providers should following the programme standards. According to the programme standards criteria for the TCM education include fundamental courses in TCM, western medicine, and clinical practice et al unfortunately, there is a shortage of neurology education components in current TCM educational system in Malaysia.

The field of neurology is widely recognized as the most challenging specialty among medical and TCM students, as well as non-specialist healthcare professionals, both during their medical training and beyond, on a global scale.^[1]^ This perception often leads students and healthcare professionals to view neurology as less intriguing, especially considering that many neurological conditions are often encountered first by general practitioners and other healthcare providers.^[2]^ Consequently, this lack of interest may deter aspiring medical and TCM students or general practitioners from pursuing a career in neurology. To address this issue, it is essential to enhance the neurology education of TCM students by emphasizing core knowledge and refining their clinical practical skills, which could significantly improve neurology disease management.^[3]^

In this regard, the implementation of problem-based learning (PBL) methods emerges as a crucial solution to overcome the limitations of traditional teaching techniques.^[4]^ Conventional teaching in neurology suffers from various drawbacks, including: Limited Interactivity: Traditional teaching predominantly relies on lectures, textbooks, and written materials, which can hinder student engagement and interaction among peers; Struggling to Keep Pace: Neurology is a rapidly evolving field, continuously generating new research and discoveries. Unfortunately, conventional teaching methods may struggle to keep up with these latest developments; Neglecting Individual Learning Differences: Students have diverse learning styles and preferences, yet conventional teaching methods often fail to effectively cater to these individual differences, resulting in suboptimal learning outcomes for some learners.^[5]^

To address these challenges and boost interest and understanding in neurology, a shift towards PBL can offer more engaging, up-to-date, and adaptive educational experiences for aspiring medical and TCM students, as well as general practitioners. By focusing on practical problem-solving and interactive learning, this approach can better equip students to excel in the dynamic and demanding field of neurology.

PBL has been widely used in medicine fields and educational context to promote critical thinking and problem-solving in authentic learning outcomes.^[6]^ It is a pedagogical approach whereby issues are explained within a scenario to enable medical students to identify their own learning objectives,^[7]^ and involves students working in small groups through a specific problem or set of problems, with the oversight of a lecturer. The problem solving within the group generates topics for individual self-study, after that the group reconvenes to discuss the topic further.^[8]^ Lecturers act as facilitators and guides, it is the lecturers does not provide knowledge or information directly. Lecturers are encourage skills of inquiry by appropriate probing problems or questions of the students and encouraging students to develop the knowledge and critical thinking pathway to ask themselves and their peers to learn new knowledge and information.^[9]^ The PBL is recognized as a successful innovative learning method in the undergraduate medical education system, also effective in enhancing students clinical practice performance,^[10]^ and analytical skills.^[11]^

In this observational study, we conducted PBL learning method for the Neurology education in the TCM undergraduate students. We evaluated whether it can make up for the shortcomings of conventional teaching, improve students learning interesting and students basic knowledge of neurology disease management.

## 2. Methods

### 2.1. Study design and setting

The cross-sectional online survey was collected at Xiamen University Malaysia. In the 2022/04 academic semester, the course titled “Hello, Neurology” was the reformed from the conventional learning methods to the PBL methods, it is designed and approved from the external examiner, registered neurologist, and certified the PBL instructors. The School of Traditional Chinese Medicine, Xiamen University Malaysia confirmed the content validity of each course contents and carried out the interventions in total 8 sessions, each sessions conducted 2 hours for each group. The general course objectives are introduce the neurology and disease management to TCM undergraduate students.

### 2.2. Participants

The comparative study was conducted 2020/02 and 2020/04 intake the year 3 TCM undergraduate students of the Xiamen University Malaysia. A total of 86 were enrolled in this study. They were randomly divided into conventional learning group and PBL group. Students who missed more than 1 session of the course and those who did not completed the questionnaires in the evaluation periods were excluded from the study (n = 0).

All lecturers were trained and certified the PBL senior lecturers/Assistant Professor/Associate Professor with registered teaching permit holders in the Ministry of Higher Education Malaysia.

### 2.3. Ethics approval and consent to participate

This study was approved by the School of Traditional Chinese Medicine Research Ethics Committee of the Xiamen University Malaysia (no. REC-2208.01). All participants completed informed consent form that included the study purpose before start of the study. All participants were reassured of the confidentiality and the right to withdraw at any time during the study. All methods were carried out in accordance with the institutional guidelines and regulations, and written informed consent was obtained from all participants.

### 2.4. Training of the PBL lecturers

Between the November 2021 and February 2022, the Xiamen University Malaysia carried out 2 day introductory courses on PBL principles for 12 academic staffs to involved in curriculum development, and on willingness to learn and hands-on teaching practice the PBL approach. The training learning objectives were to introduction, application, development and setting the PBL approach and simulate teaching practice, and tutorial sessions. During the training, academic staffs to understand and demonstrated PBL context and curriculum development using the focusing small group discussion. At the same time discussed the learning materials and evaluation questionnaire tools.

### 2.5. Conventional and PBL teaching

We chose neurology disease and management contents as the topic for applying the conventional and PBL teaching approach in this study.

By selecting a specific topic, such as neurology disease and management, the researchers can focus their study on a well-defined area, allowing for more accurate and reliable comparisons between the 2 teaching approaches. This targeted approach ensures that the content taught and the learning outcomes assessed are consistent, enabling a direct comparison of the effects of conventional teaching and PBL on the same subject matter. Furthermore, neurology disease and management is a complex and challenging subject, making it an appropriate choice for testing the effectiveness of different teaching methods.^[12]^ The study can assess how well students grasp the material, retain information, apply critical thinking skills, and problem-solving abilities using both conventional and PBL approaches. The selecting neurology disease and management contents as the topic for the study is a suitable decision, as it allows for a focused comparison between conventional and PBL teaching approaches, providing valuable insights into their respective effects on student learning outcomes.^[13]^

The faculty was conducted the conventional group was arranged as following:

The course lecturers prepared lecture slides and Supplementary materials provide the students. The student were received about the disease definition, epidemiology, pathogenesis, differential diagnosis, disease management and prevention was carried out. It is course lecturer provided a thorough explanation of the theoretical medical knowledge, and the students answered the questions and made suggestions.

The PBL groups was arranged as following:

Firstly, the course lecturers conducted the disease definition and disease management and put forward to representative and enlightening questions according to the characteristics of the neurologic disorders.

Secondly, during the small group discussions, the students under the PBL lecturers monitoring and guidance. The students were encouraged to raise relevant clinical questions and problems, and searched the answers on the Internet, consult main reference books, and connect the university library database or other relevant materials. This process has been described as the 7 classical steps of PBL. Knowledgeable and understand the situation and clarify terminology; Identify the questions and problems; Suggest possible causes; Connect questions and problems and causes; Decide what type of information is needed; Obtain relevant information; Apply the information. Depending on the complexity of the questions and problems, additional mentoring may be required as the group narrows the possible solutions.^[14]^

Thirdly, the student representative from each group to demonstrate to review the main points from the lessons, posed their group problems and questions and its relevant solving, and Q&A about unsolved problems and questions.

Finally, the PBL lecturers summarized the class and went over the problems and questions.

## 3. Outcome measurement

### 3.1. The theoretical and clinical practical skill examinations

All participants received theoretical and clinical practical skill examinations before and after completed learning. The both learning contents consisted of comprehensive theoretical and clinical training, culminating in theoretical and clinical practical skills examinations. These assessments were conducted before and after the completion of the learning to gauge participants progress and competency in neurology.

The theoretical written examination:

The theoretical written examination was administered to all participants before and after they completed the learning. This examination aimed to assess participants understanding of fundamental concepts related to neurology disorders. It included the following contents:

1. Disease definitions: participants were required to demonstrate their knowledge of various neurology disorders, including their etiology, pathophysiology and classification.
2. Clinical symptoms: participants were tested on their ability to identify and describe the clinical manifestations associated with different neurology disorders.
3. Management: this sections assessed participants knowledge of appropriate medical interventions, therapies, and management strategies for specific neurology disorders.
4. Prevention: participants were evaluated on their understanding of preventive measures and strategies to mitigate the risk of neurology disorders.

Clinical practical skill examination:

In addition to the theoretical written examination, participants also underwent a clinical practical skill examination before and after completing the learning. This examination aimed to evaluate participants ability to apply their theoretical knowledge in real-world scenarios. The clinical practical skill examination included the following components:

1. Diagnosis and differential diagnosis: participants were presented with patient care studies representing various neurology disorders. They were required to diagnose the condition accurately and distinguish it from other similar disorders through clinical reasoning and analysis.
2. Selection of treatment procedures: this section assessed participants competence in choosing appropriate treatment plans and procedures based on the diagnosed neurology disorders.

### 3.2. Scoring and assessment

Participants scores were calculated based on the number of correct answers they provided in each examination. The maximum achievable score for both the theoretical and clinical practical skills examinations was 10 points. The scores obtained in the pre-assessment were compared to those of the post-assessment to determine the participants improvement and overall progress throughout the learning.

### 3.3. The satisfaction teaching level

Focusing on the investigation of satisfaction with teaching methods, the teaching method effect was evaluated through a feedback survey. The satisfaction with the teaching method included high-level satisfaction (above 80 points), general satisfaction (60–80 points), and dissatisfaction (<60 points). Then, the satisfaction degree was calculated according to the formula: satisfaction degree = high-level satisfaction rate + general satisfaction rate.^[15]^

### 3.4. The students perspectives and self-learning competence

A comprehensive anonymous survey was carried out at the conclusion of the course, focusing on the evaluation of teaching methods. The survey encompassed all participants, and its questionnaires aimed to gather insights into the students perspectives and their self-perceived competence within the PBL and conventional learning groups.

The survey findings provided valuable data on various aspects of the student experiences. These aspects included scores for knowledge and understanding, cognitive abilities, lecturer-student interactions, communication skills, clinical practical skills, self-learning skills, teamwork abilities, leadership skills, as well as ethics and professionalism. The survey results yielded valuable and comprehensive data regarding several key aspects of the student academic experiences. These aspects encompassed a wide range of factors, such as:

Knowledge and understanding: this category gauged the students grasp and comprehension of the course material and subject matter.

Cognitive abilities: it assessed the students cognitive prowess, including critical thinking, problem-solving, and analytical skills.

Lecturer-student interactions: this aspect focused on the quality of interactions between students and their lecturers, examining factors such as approachability, responsiveness, and effectiveness of communication.

Communication skills: this category explored the students proficiency in expressing ideas, both orally and in writing, as well as their ability to effectively communicate with peers and instructors.

Clinical practical skills: it evaluated the students competence in applying theoretical knowledge to practical situations, particularly in clinical settings.

Self-Learning skills: this aspect assessed the students ability to independently acquire knowledge and skills through self-directed learning methods.

Teamwork abilities: it examined how well students collaborated with others, their contribution to group tasks, and their aptitude for working in a team-oriented environment.

Leadership skills: this category gauged the students potential to take charge and guide others effectively, exhibiting leadership qualities within academic and professional contexts.

Ethics and professionalism: it focused on evaluating the students adherence to ethical standards and professionalism in their academic pursuits and interactions.

The survey encompassed a broad spectrum of vital aspects concerning the efficacy and influence of diverse teaching approaches, providing an all-encompassing assessment of students perspectives and self-assessed proficiency in various fundamental domains. The questionnaires used in this study were adopted from previous articles.^[16,17]^ Participants responded to the questions using a 5-point Likert scale, where 1 denoted the lowest level of agreement and 5 indicated the highest. For a detailed list of questions in each category (Supplemental 1, http://links.lww.com/MD/K26).

### 3.5. The students perceptions of learning environment survey

The students perceptions of learning environment survey concerning the teaching methods by PBL and conventional teaching were sought by the use of a survey questionnaires was adopted dundee ready educational environment measure (DREEM), it was designed to measure the educational environment specifically for medicine and other health professionals.^[18]^ The primary purpose of the DREEM is to assess the quality of the educational environment within a particular institution or program, from the perspective of the students. The DREEM questionnaire is designed to gather students perceptions and attitudes toward various aspects of their learning environment. It is cover 5 key domains related to the educational environment:

Perception of learning: this domain explores how students perceive the effectiveness of teaching and learning methods, including lectures, practical sessions, and clinical teaching.

Perception of teachers: this domain assesses students views on the teaching faculty, including their approachability, enthusiasm, and supportiveness.

Academic self-perceptions: this domain focuses on students self-confidence in their academic abilities and their perception of the learning atmosphere.

Perception of atmosphere: this domain looks at the social and emotional aspects of the educational environment, including students feelings of safety and their interaction with peers.

Social self-perception: this domain evaluates students perceptions of the social environment and their sense of belonging within the institution.

Interpreting the DREEM results can provide valuable insights into the strengths and weaknesses of the educational environment from the students perspective. Higher DREEM scores indicate a more positive perception of the educational environment, suggesting a more conducive and supportive learning atmosphere. Conversely, lower DREEM scores may indicate areas of concern that need improvement to enhance the overall learning experience.^[19]^ Educational institutions and program administrators often use the DREEM as a formative assessment tool, which means they regularly administer it to identify areas that require attention and make necessary improvements in the educational environment to optimize the learning experience for students.^[20]^ Additionally, DREEM can be used for research purposes to compare the educational environments across different institutions or programs and track changes over time.

There were fifty items in the questionnaire to assess for the students perceptions of learning environment. Each relates to 1 of 5 themes: Students perceptions of learning (12 items); Students perception of teachers (11 items); Students academic self-perceptions (8 items); Students perceptions of the learning atmosphere (1 items); Students social self-perceptions (7 items). Responses to the items are scored from 4 to 0, and 0 represented strongly disagree and 4 represented strongly agree. However, reverse scoring was required for items 4, 8, 9, 17, 25, 35, 39, 48, and 50. The DREEM score is calculated between 0 to maximum score of 200.^[21,22]^ According to for an international association for medical education guideline interpreted, a score of 51 to 100 indicates “plenty of problems,” while a score of 101 to 150 is “more positive than negative” and score of 100 is interpreted as “considerable ambivalence by students and needs to be improve.” Items with mean scores of less than 2 should be closely considered. Except for negative items mentioned, a higher score means better interpretation.^[23,24]^

### 3.6. Statistical methods

The data collection and statistical analyzed using the SPSS software version 16.0 for Windows (IBM). All data are shown as mean ± standard deviation (SD). Applying the independent sample *t* test was used to compare results between the PBL and conventional groups. Statistical significance was inferred at *P* values < .05.

## 4. Result

**Figure 1.**
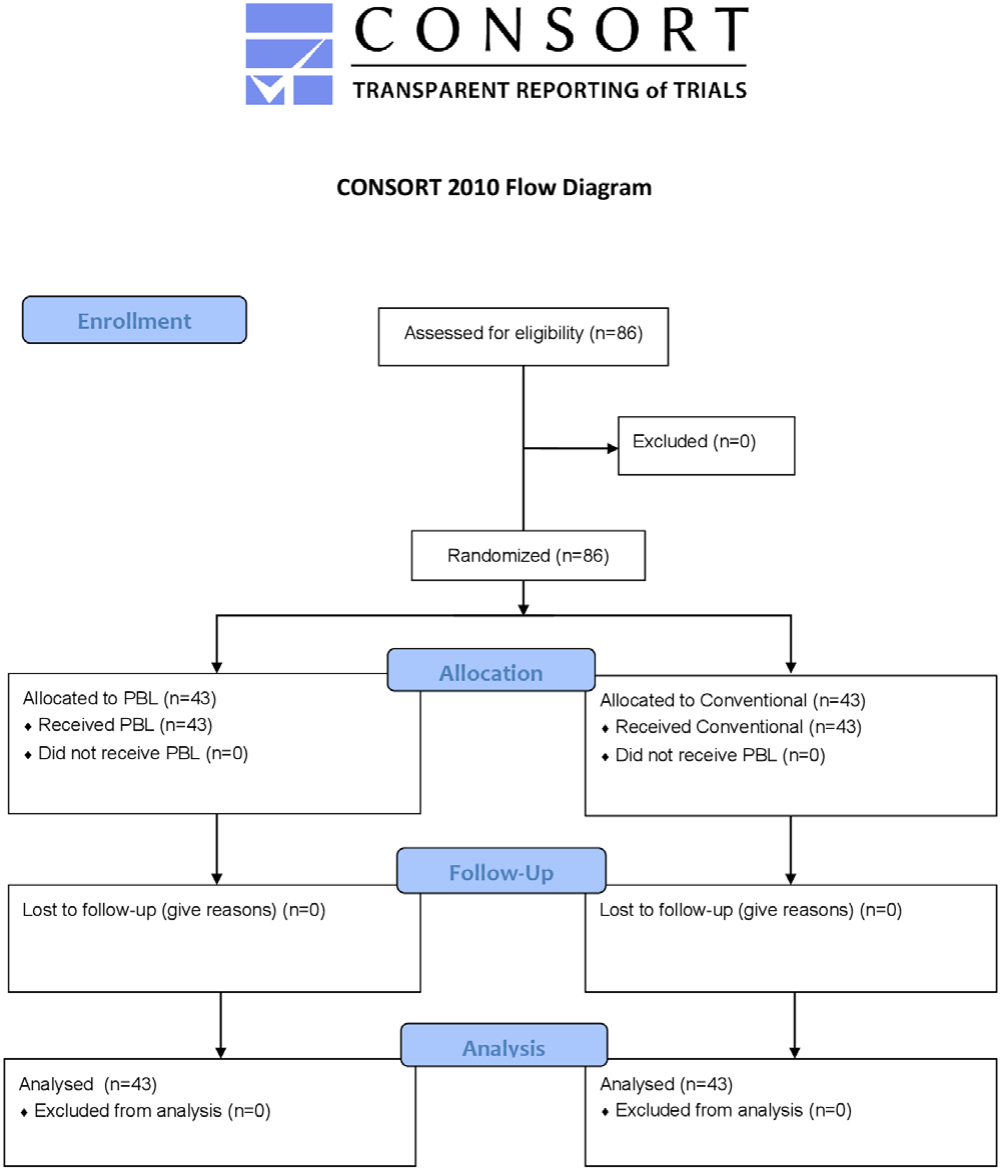
CONSORT study flow diagrams.

Eighty-six participants were present at the initial session, and all eighty-six entered the study and were equally allocated into the conventional and PBL groups, including forty-three participants in each group. All eighty-six participants completed the theoretical and clinical practical examinations and questionnaires. Figure 1 shown the CONSORT study flow diagrams for this study.

### 4.1. Participants socio-demographics

A total 86 participants were enrolled in this study. They were randomly divided into PBL group (n = 43) and conventional group (n = 43). There were 20 (46.5%) male and 23 (53.5%) female participants in the PBL group, in conventional group were 24 (55.8%) male and 19 (44.2%) female participants. There are 10 (23.2%) in the PBL and 8 (18.6%) in the conventional groups who were scholarship holders. Total 36 (83.7%) Malaysian and 7 (16.3%) Chinese in the PBL group, There are 38 (88.3%) Malaysian and 5 (11.7%) Chinese in the conventional group. The average age of PBL group were 22.7 ± 2.1 and conventional group were 22.6 ± 1.8 years respectively. There was no significant difference in age, gender, scholarship, and nationality (*P* > .05, data shown the Table 1).

**Table 1:** Socio-demographic characteristics of the participants in the study (n = 86).

	PBL (n = 43)	Conventional (n = 43)	*P* value
Gender			.716
Male	20 (46.5%)	24 (55.8%)	
Female	23 (53.5%)	19 (44.2%)	
Scholarship			.629
Yes	10 (23.2%)	8 (18.6%)	
No	33 (76.8%)	35 (81.4$)	
Nationality			.896
Malaysian	36 (83.7%)	38 (88.3%)	
Chinese	7 (16.3%)	5 (11.7%)	
Age (yr, Mean ± SD)	22.7 ± 2.1	22.6 ± 1.8	.901

PBL = problem based learning.

### 4.2. The comparison of theoretical and clinical practice examination scores between the PBL and conventional groups

The Table 2 shown the before and after Theoretical and Clinical practical skill examination scores between the PBL and conventional groups.

**Table 2:** Comparison of the theoretical and clinical practice examination scores between the PBL and conventional groups.

	PBL (n = 43)	Conventional (n = 43)	*P* value
Before			
Theoretical examination	3.27 ± 1.65	3.39 ± 1.51	.162
Clinical practical skill examination	3.86 ± 1.83	4.05 ± 1.56	.396
After			
Theoretical examination	8.35 ± 1.39*	6.01 ± 1.46	.019
Clinical practical skill examination	7.79 ± 1.52*	6.98 ± 1.23	.024

Values represent mean ± SD.

PBL = problem based learning.

* Indicates a statistical difference from the conventional group (*P* < .05).

In the conventional group, the before Theoretical examination score were 3.39 ± 1.51 and Clinical practical skill examination score were 4.05 ± 1.56; after Theoretical examination score were 6.01 ± 1.46 and Clinical practical skill examination score were 6.98 ± 1.23. In the PBL group, the before Theoretical examination score were 3.27 ± 1.65 and Clinical practical skill examination score were 3.86 ± 1.83; after Theoretical examination score were 8.35 ± 1.39 and Clinical practical skill examination score were 7.79 ± 1.52 respectively. There are no statistical significance in pre Theoretical examination and Clinical practical skill examination scores both groups (*P* > .05). The after Theoretical examination and Clinical practical skill examination scores in the PBL group were significantly higher than conventional group (*P* < .05).

### 4.3. The satisfaction teaching level of TCM students with different teaching groups

Comparing the satisfaction teaching level of TCM students in the PBL and conventional groups, we found the overall satisfaction teaching level of the PBL group was significantly higher than conventional group (*P* < .05). The data shown in the Table 3.

**Table 3:** The satisfaction teaching level of traditional Chinese medicine (TCM) students with different teaching methods

	Satisfaction	General	Dissatisfaction	Satisfaction degree	*P* value
PBL (n = 43)	38	3	2	95%*	.038
Conventional (n = 43)	18	16	9	79%	

PBL = problem based learning.

* Indicates a significant difference from the conventional group (*P* < .05).

### 4.4. The questionnaire survey scores between the PBL and conventional groups

We conducted after course survey scores concerning to students perspectives and self-learning competence in the PBL and conventional groups. We found the cognitive, lecturer-student interaction, communication skills, clinical practical skills, self-learning skills, teamwork skills, and leadership skills were significantly higher in the PBL group than in the conventional group. The results shown in the Table 4.

**Table 4:** Questionnaire survey scores between the PBL and Conventional groups.

	PBL (n = 43)	Conventional (n = 43)	*P* value
Knowledge and understanding	4.12 ± 0.87	4.01 ± 0.56	.062
Cognitive	4.02 ± 0.28*	3.03 ± 0.17	.015
Lecturer-student interaction	4.99 ± 0.82*	2.94 ± 0.74	.024
Communication skills	4.89 ± 0.76*	2.85 ± 0.81	.014
Clinical practical skills	4.78 ± 0.68*	2.57 ± 0.81	.012
Self-learning skills	4.07 ± 0.79*	2.42 ± 0.63	.017
Teamwork skills	3.96 ± 0.82*	2.40 ± 0.81	.031
Leadership skills	4.08 ± 0.51*	2.51 ± 0.65	.029
Ethics and professionalism	4.13 ± 0.91	4.08 ± 0.82	.071

Values represent mean ± SD.

PBL = problem based learning.

* Indicates a statistical difference from the conventional group (*P* < .05).

### 4.5. The comparison of DREEM scores between the PBL and conventional groups

The Table 5 shown the total DREEM scores for the PBL group was significantly higher than the conventional group. We found the Students Perception of Learning, Students Perception of Teachers, Students Academic Self-Perception, and Students Social Self-Perception scores were significantly higher in the PBL group than the conventional group. However, Students Perception of Atmosphere score were not significantly difference between the PBL and conventional groups.

**Table 5:** The comparison of DREEM scores between the PBL and conventional groups

DREEM	Max. Score	PBL (n = 43)	Conventional (n = 43)	*P* value
SPL	48	31.37 ± 7.1*	27.39 ± 7.3	.012
SPT	44	27.07 ± 5.9*	24.78 ± 5.6	.017
SAP	43	20.44 ± 5.5*	18.68 ± 5.1	.015
SPA	48	27.72 ± 8.8	27.47 ± 6.8	.063
SSP	28	15.88 ± 4.9*	10.55 ± 4.1	.023
Total DREEM score	200	122.58 ± 25.6*	115.91 ± 22.7	.019

Values represent mean ± SD.

DREEM = dundee ready education environment, PBL = problem based learning, SAP = students academic self-perception, SPA = students perception of atmosphere, SPL = students perception of learning, SPT = students perception of teachers, SSP = students social self-perception.

* Indicates a statistical difference from the conventional group (*P* < .05).

## 5. Discussion

Neurology is a branch of general medicine dealing with disorders of the nervous system, is considered one of the most intimidating and complicated fields of the medical sciences by medical students and general practitioners.^[25]^ The neurology education is that it is an evolving field with new developments and advancements happening all the time. Neurology education generally consists of a combination of didactic lecturers, clinical studies, and research experiences. Understanding and being well-trained in neurology could spend considerable time and effort, in fact, it is a lack of systematic and comprehensive neurology education for undergraduate students and continuing education programme for general practitioners or other related healthcare professionals, who wrongly understand neurology disorders and management.^[26]^ One issues in neurology education is the shortage of well-trained neurologists and related healthcare professionals. There is an increasing demand for neurologists or related healthcare professionals as the population ages and neurological disorders become more prevalent. However, there are not enough undergraduate students choosing neurology as their specialty and future job skills, which creates a shortage of qualified healthcare professionals. Another issue in neurology education is the need for more emphasis on interdisciplinary training.^[27]^ Neurological disorders often require a multidisciplinary approach, with specialists from different fields working together to provide the best medical health care. As such, neurology education needs to incorporate training in areas such as psychiatry, neurosurgery, rehabilitation medicine, and integrative medicine.

TCM education in Malaysia is divided into 2 concepts—academic and skill-based education. The main educational aim of this is the quality control of higher education providers that provide high-impact academic education. As the higher education provider of TCM to overcome the challenges associated with the need to improve the resources required to ensure the quality of high-impact education.^[28,29]^ Moreover, the TCM students stipulate stronger integrative medical concepts to completing the regular curriculum. Although this provision has not yet been implemented, such strong integrative medical concepts also advanced the level of education for the new generation of TCM practitioners who are knowledgeable in biomedical sciences, western medical sciences, and well-versed in TCM and at the same time adequately educated medical law, medical ethics, professionalism, and humanities.^[30]^

PBL education starting from learning is a collaborative process anchored in student groups. The principles further state how medical students are responsible for their own personal learning while being supported by one or more lecturers. It is also emphasized that problems must be exemplary and scientific. Problems must therefore reflect conditions realistic and authentic within a medical academic field or relevant to a profession.^[31]^ Therefore, PBL education is concerned with the future professions of medical students and promotes metacognitive skills by having medical students engage with authentic and complex problems. Thus, PBL is reflected in competencies such as the ability to self-learning, to collaborate with group members, and to initiate and organize discussion when encountering complex real-life problems during clinical practice.^[32]^

In conclusion, the observational study aimed to compare the effectiveness of PBL and conventional teaching methods in neurology education. The results indicated significant differences in the post-examination scores between the 2 groups, favoring the PBL group. After the intervention, the PBL group demonstrated higher theoretical and clinical practical skill examination scores compared to the conventional group.

Furthermore, the overall satisfaction teaching level of TCM students in the PBL group was significantly higher than that of the conventional group, as evidenced by the questionnaire survey scores. The PBL group outperformed the conventional group in various aspects, including cognitive abilities, lecturer-student interaction, communication skills, clinical practical skills, self-learning skills, teamwork skills, and leadership skills.

Additionally, the total DREEM scores, representing students perceptions of the learning environment, were significantly higher in the PBL group than in the conventional group. Students in the PBL group perceived their learning, teachers, academic self-perception, and social self-perception more positively compared to those in the conventional group. However, there were no significant differences in the Students Perception of Atmosphere score between the 2 groups.

In summary, this study provides evidence that PBL is a more effective and satisfying teaching approach for TCM students compared to conventional methods in neurology education. The findings suggest that PBL enhances students theoretical and practical skills, cognitive abilities, and overall learning experience. Implementing PBL in neurology education can potentially lead to better-prepared and more competent TCM students in the future.

## 5. Conclusion

On the basis of the results of the present study, it can be concluded that the PBL teaching method is preferable for neurology education in TCM undergraduate students over the conventional teaching method. The PBL teaching method offers substantial benefits in supporting and stimulating the learning interest of TCM undergraduate students, while also enhancing their logical thinking, problem-solving skills, and innovative solutions. This effective educational approach fosters a profound understanding of the subject matter, cultivating essential competencies like problem-solving, critical thinking, and collaborative abilities. Moreover, PBL empowers undergraduate students to apply their knowledge to real-world scenarios, infusing their learning with greater significance and practicality.

In addition to its academic advantages, PBL plays a crucial role in equipping undergraduate students with essential professional skills like communication, teamwork, and leadership, which hold immense value in the medical career field. Through PBL, students are encouraged to approach challenges with a proactive and multidimensional mindset, enabling them to excel not only academically but also in their future medical practices.

This study highlighted the PBL teaching method is a highly valued educational format and has been broadly applied in neurology education, such as for the retention and application of neurology disease and its clinical management knowledge. PBL encourages undergraduate students to actively engage with a problem, research possible solutions, and work collaborate to develop a deeper understanding of the topic.

There are still some limitations to consider, the current study can’t conduct double-blind research and compared other traditional medical education teaching methods, such as case-based learning methods. PBL may not be suitable for all undergraduate students, as it requires a certain level of self-directed learning skills and may not be effective for undergraduate students who prefer a more structured approach to learning.^[33]^ Additionally, the effectiveness of PBL may depend on the quality of the problem presented and the facilitation of the learning process.^[34,35]^ Finally, there is a lack of standardized assessment methods for PBL, which makes it difficult to compare outcomes across different studies.^[36]^ Despite these limitation, PBL remains a valuable approach to promoting critical thinking, problem-solving, and collaboration skills, and further research can help to address these limitations and optimize its implementation.

## Acknowledgments

The authors thanks to all the TCM undergraduate students who volunteered to participate in the study, and Prof Xin Gang, Department of Microbiology and Immunology, Shantou University, China to conduct the PBL training.

## Author contributions

**Data curation:** Yun Jin Kim.

**Formal analysis:** Yun Jin Kim.

**Funding acquisition:** Yun Jin Kim.

**Investigation:** Yun Jin Kim.

**Methodology:** Yun Jin Kim.

**Project administration:** Yun Jin Kim.

**Resources:** Yun Jin Kim.

**Supervision:** Yun Jin Kim.

**Validation:** Yun Jin Kim.

**Visualization:** Yun Jin Kim.

**Writing – original draft:** Yun Jin Kim.

**Writing – review & editing:** Yun Jin Kim.

## Supplementary Material


