# Supplementary material for: The PBL teaching method in neurology education in the traditional Chinese medicine undergraduate students: An observational study
Source: Medicine (Baltimore). 2023 Sep 29;102(39):e35143. doi: 10.1097/MD.0000000000035143 (PMC10545269; doi:10.1097/MD.0000000000035143)
Supplement: Supplementary file 1 [file medi-102-e35143-s001.docx]

**Supplement 1. Questions for students’ perspectives and self-learning competence**

|  | Question | 1 | 2 | 3 | 4 | 5 |
| --- | --- | --- | --- | --- | --- | --- |
| Knowledge and Understanding | Capable of imparting fundamental knowledge. |  |  |  |  |  |
| Cognitive | Enhances academic performance and overall learning experience in a positive manner. |  |  |  |  |  |
| Lecturer-Student interaction | Share your overall experience regarding interactions between lecturers and students in your current learning environment. |  |  |  |  |  |
| Communication skills | Are you comfortable expressing your ideas and opinions during class discussions? |  |  |  |  |  |
| Clinical Practical skills | Exhibit clinical practice skills. |  |  |  |  |  |
| Self-learning skills | Are you actively involved in self-learning beyond the classroom? |  |  |  |  |  |
| Teamwork skills | How significant do you consider teamwork skills during the learning process? |  |  |  |  |  |
| Leadership skills | Displayed active helpfulness when other students faced difficulties. |  |  |  |  |  |
| Ethics and Professionalism | Do you believe ethics and professionalism are essential in education and future career pursuits after completing your studies? |  |  |  |  |  |
